# Experimental characterization of extreme events of inertial dissipation in a turbulent swirling flow

**DOI:** 10.1038/ncomms12466

**Published:** 2016-08-31

**Authors:** E. -W. Saw, D. Kuzzay, D. Faranda, A. Guittonneau, F. Daviaud, C. Wiertel-Gasquet, V. Padilla, B. Dubrulle

**Affiliations:** 1 SPEC, CEA, CNRS, Université Paris Saclay, CEA Saclay, 91191 Gif-sur-Yvette, France; 2 LSCE, IPSL, CEA-CNRS-UVSQ, Université Paris-Saclay, 91191 Gif-sur-Yvette, France; 3 ENS Lyon, 46 Allée d'Italie, F-69007 Lyon, France

## Abstract

The three-dimensional incompressible Navier–Stokes equations, which describe the motion of many fluids, are the cornerstones of many physical and engineering sciences. However, it is still unclear whether they are mathematically well posed, that is, whether their solutions remain regular over time or develop singularities. Even though it was shown that singularities, if exist, could only be rare events, they may induce additional energy dissipation by inertial means. Here, using measurements at the dissipative scale of an axisymmetric turbulent flow, we report estimates of such inertial energy dissipation and identify local events of extreme values. We characterize the topology of these extreme events and identify several main types. Most of them appear as fronts separating regions of distinct velocities, whereas events corresponding to focusing spirals, jets and cusps are also found. Our results highlight the non-triviality of turbulent flows at sub-Kolmogorov scales as possible footprints of singularities of the Navier–Stokes equation.

## Abstract (web summary)

The classical description of viscous turbulent flows is based on a formulation of Navier-Stokes equations which assumes its solutions to remain smooth at all times. Saw *et al*. characterize velocity fields in experimental turbulent flows at dissipative scale, and link the results to the singularities.

## Graphical abstract

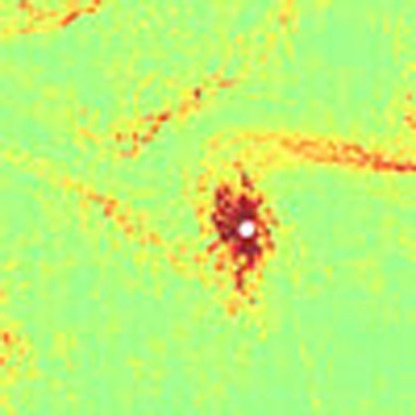

About 500 years ago, Leonardo Da Vinci published what appears to be one of the first detailed experimental account of vortices in water. It then took three centuries to establish the fundamental equations describing the dynamics of water, now known as the incompressible Navier–Stokes equations (INSE):


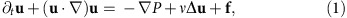



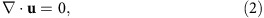


where **u** is the *d*-dimensional velocity field, *P* the kinematic pressure, **f** a forcing and *v* the kinematic viscosity. A natural control parameter of the INSE is the Reynolds number *Re*=*UL*/*v*, built using a characteristic length *L* and velocity *U*. The INSE are the cornerstones of many physical and engineering sciences, and are routinely used in numerical simulations^1^,^2^,^3^,^4^. From a mathematical point of view, however, it is still unclear whether the INSE are a well-posed problem in three dimensions, that is, whether their solutions remain regular over sufficient large time or develop singularities. This motivated their inclusion in the AMS Clay Millennium Prize list^5^.

Historically, the search for singularities in INSE was initiated by Leray^6^ who introduced the notion of weak solutions (that is, in the sense of distribution). This notion was used to prove that the mathematical singular set has a one-dimensional Haussdorff measure equals to zero in spacetime^7^,^8^. Therefore, if these singularities exist, they must be extremely localized events in space and time. This makes their direct detection an outstanding problem. For some times, the best evidence of their existence was provided by the observation that the energy dissipation rate in turbulent flows tends to a constant at large Reynolds numbers^9^. This observation is at the core of the 1941 Kolmogorov theory of turbulence^10^ and was interpreted by Onsager^11^ as the signature of singularities with local scaling exponent *h*=1/3. Later, it was conjectured^12^ that the singularities are organized into a multifractal set. Analysis of measurements of three-dimensional numerical or one-dimensional experimental velocity fields showed that the data are compatible with the multifractal picture, with a most probable *h* close to 1/3 (refs 13, 14). However, this analysis could not reveal any information on the space-time statistics of possible singularities.

A major breakthrough was achieved when Duchon and Robert^15^ derived a detailed energy balance for weak solutions of INSE and computed the contribution stemming from an eventual lack of smoothness. They show that it can be lumped into a single term *D*(**u**), which quantifies the ‘inertial' energy dissipation, that is, the energy dissipated by non-viscous means. They define dissipative weak solutions of Navier–Stokes equations as those with *D*(**u**)⩾0, the equality being only achieved for smooth-enough solutions (corresponding to a local scaling exponent *h*>1/3). Later, Eyink^16^ proved the existence of a like-wise non-zero rate of velocity circulation decay 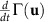, produced by singularities. These mathematical results are obtained in the limit of vanishing spatial scales, so that their direct application to experimental or numerical flows is problematic. In such cases, one can only expect to be able to measure coarse-grained quantities, *D*_ℓ_(**u**) and 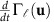, at a scale ℓ dictated by experimental or numerical constraints^17^. In that respect, a special role is played by the so-called dissipative scale ℓ=*η*, as it is traditionally expected to be the scale at which all injected energy is converted into viscous dissipation, and the flow is regularized by viscosity. For example, it is at this scale that numerical simulations are usually truncated, or experimental velocity gradients estimated. On the road to the mathematical limit ℓ→0, it seems interesting to study the properties of *D*_ℓ_(**u**) and 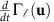 down to the dissipative scale.

The purpose of the present study is to use high spatial resolution measurements of the velocity field in experiments of turbulent swirling flow (see ‘Methods' for more on this choice) to compute *D*_*ℓ*_(**u**) and 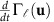 down to dissipative scales. We show that they are very intermittent in space and time, and provide the first experimental attempt at characterization of isolated extreme events of inertial dissipation. By characterizing the local topology of these events, we find that most of them appear as fronts separating regions of distinct velocities, whereas some correspond to focusing spirals, jets and cusps. Our results highlight the non-triviality of turbulent flows at sub-Kolmogorov scales as possible footprints of singularities of the Navier–Stokes equation.

## Results

### Relevant hydrodynamic parameters in von Kármán swirling flows

Details on the setup can be found in the Methods section. We vary the rotating frequency (*F*) of the impellers that drive the flow and use different mixture of glycerol/water, to vary the viscosity of the working fluid, and thus the Reynolds number *Re*=*2πFR*^2^/*v*, (where *R* is the radius of the impellers). Monitoring the torques *C*_1_ and *C*_2_ applied to each impeller, we obtain the energy injection rate (per unit mass of fluid) as:


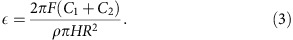


where *ρ* is the fluid's mass density and *H* the distance between the impellers. From this, we can compute the Kolmogorov dissipative scale as 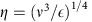.

In a statistically stationary regime, the energy input must balance the rate of energy dissipated within the flow. This has been checked in a scale 4:1 version of our experiment in Helium, using precise calorimetric measurements^18^. Previous global dissipation measurements have shown that the dimensionless energy dissipation rate saturates at large *Re* towards a value that depends on the impellers and the mean flow geometry^19^ (more details in Supplementary Note 1). This property allows us to determine the threshold for the onset of fully developed turbulence as *Re*≈3,500. This also corresponds to the threshold where non-dimensional velocity fluctuations become independent of the Reynolds number^20^.

Here we present three cases of the experiments. Case A: 100% glycerol, where the flow is laminar; Case B: 59% glycerol by volume in water, where the flow is fully turbulent; and Case C: pure water (0% glycerol), where the flow is also fully turbulent. Table 1 lists the various parameters of the cases.

### Velocity measurements and average quantities

Local velocity measurements are performed with a stereoscopic particle image velocimetry system (SPIV), providing the radial, axial and azimuthal velocity components on a meridional plane of the flow through a time series of 30,000 independent time samples. In the sequel, we work with dimensionless quantities, using *R* as the unit of length, and (2*πF*)^−1^ as the unit of time. As shown in Kuzzay *et al*.^17^, SPIV data are sufficient to detect events where *D*_*ℓ*_(**u**) takes extreme values. Essentially, it was shown, through mathematical considerations and application on experimental data that SPIV is able to detect extreme events that have components intercepting the measurement plane, and that any such events detected via SPIV is also present when volumetric three-dimensional data are considered^17^. The detection method is based on evaluating two functions of *δ***u**(ℓ), the velocity increment over a distance ℓ: (i) the inertial (non-viscous) energy dissipation rate *D*_ℓ_(**u**) and (ii) the local circulation production rate 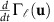 (see ‘Methods' for detailed expression). If these events are connected to singularities in the flow, they can be characterized by a local exponent *h*<1 via *δ*u∼ℓ^*h*^; these two functions should behave in the limit ℓ→0 like 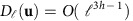 and 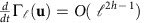 (see refs 11, 16). Previous studies based on multifractal analysis indicate that the most probable exponent is close to *h*=1/3 (ref. 13). This corresponds to a constant bound for *D*_ℓ_(**u**) as *Re*→∞. On the other hand, for stronger events with *h*<1/3, both *D*_ℓ_(**u**) and 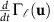 may diverge at small scales. Formally, the spatial resolution of PIV measurement is twice the grid spacing *δx*, which depends on the cameras resolution, the field of view and the size of the windows used for velocity reconstruction. In the sequel, we use 2 M-pixel cameras and two different zooms, to get measurements at *δx=*3.4 mm, for a field of view covering the whole experimental setup obtained through 32 × 32 pixel windows and *δx=*0.24 mm for a field of view zoomed on a 4 × 4 cm^2^ zone at the centre of the experiment, and reconstructed with 16 × 16 pixel windows. Table 1 summarizes the parameters corresponding to the three different cases. We see that the dissipative scale *η* is resolved for case A and B, but not for case C.

### Energy dissipation and circulation production rates

**Figure 1:**
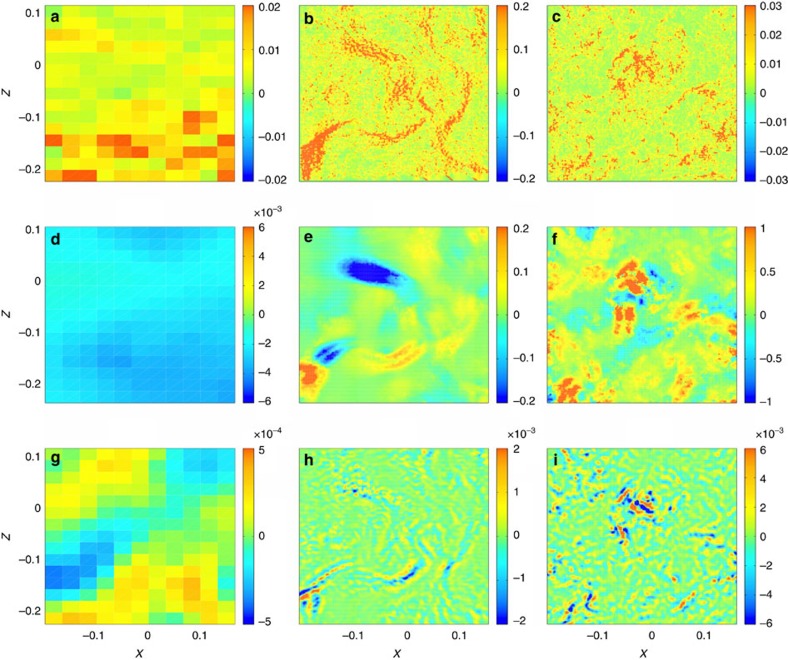
Coarse-grained energy dissipation and velocity circulation decay. Maps of the coarse-grained viscous energy dissipation 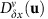 (**a** to **c**), the coarse-grained inertial energy dissipation *D*_*δx*_(u) (**d**–**f**) and the coarse-grained rate of velocity circulation decay 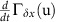 (**g**–**i**) for the three cases described in Table 1. Figures for case A are on the left panels (**a**,**d**,**g**), B on the middle panels (**b**,**e**,**h**) and C on the right panels (**c**,**f**,**i**). All the quantities have been made dimensionless using the radius *R* of the cylinder and the angular velocity (2*πF*)^−1^ of the impellers as units of length and time. We observe that the inertial dissipation remains strong in case B and C. (**g**–**i**) A non-zero circulation rate persists down to the dissipative scale. Finally, areas of high viscous dissipation seem correlated with the location of extreme events of inertial dissipation.

To study the influence of the Reynolds number and to understand how the dissipated power is split between normal (viscous) and the estimates of inertial dissipation at various scales, we have computed the local viscous dissipation 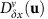, estimates of the inertial dissipation *D*_*δx*_(**u**) and the circulation production rate 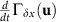 predicted by Eyink and Sreenivasan^21^, at the resolution scale of our PIV system. Maps of these three quantities for instantaneous sets of data are displayed in Fig. 1 for a region of size 4 × 3 cm^2^ located at the centre of our flow. All three cases described in Table 1 have been studied. For the three of them, we observe a smaller noise in the estimate of *D*_*δx*_(**u**) compared with 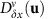. As argued in Kuzzay *et al*.^17^, this is due to the inherent smoothing procedure in the expression of *D*_ℓ_(**u**).

**Figure 2:**
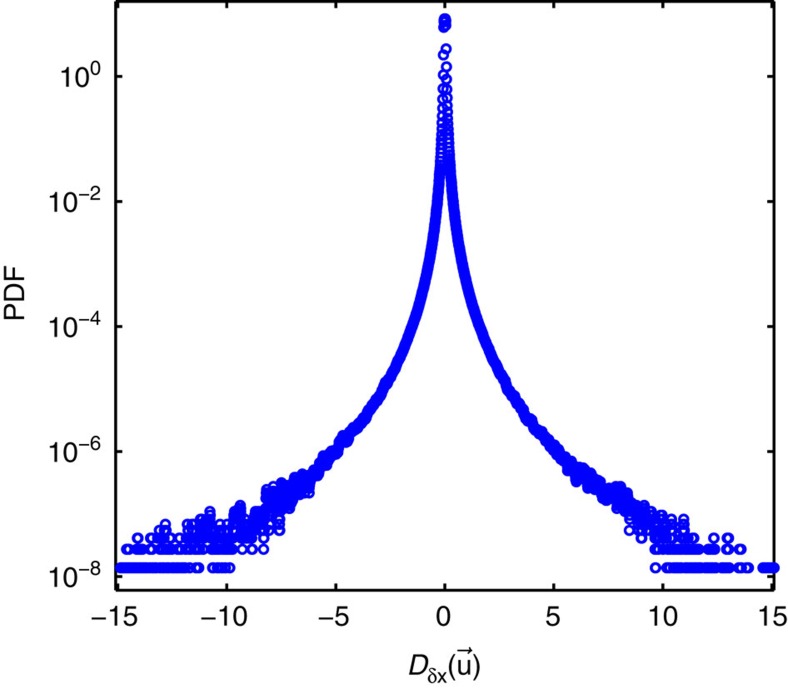
Probability density function of the estimated inertial dissipation. Probability density function (PDF) of the coarse-grained inertial dissipation *D*_*δx*_(**u**) estimated at the dissipative scale (in units of *R*^2^(2*πF*)^3^, where *R* is the radius of the cylinder and *F* the rotation frequency of the impellers), evaluated from measurements in case B. The distribution is highly non-gaussian with many events at values larger than 1,000 times the mean value.

As can be seen from Fig. 1, *D*_*δx*_(**u**) detects clear dissipation structures when the flow is fully turbulent and all scales down to the Kolmogorov scale are resolved (case B). One observes that the local inertial dissipation can be positive or negative, but on time average remains positive as reported in Table 1. This peculiar feature is parallel to the behaviour of entropy in non-equilibrium systems, where the entropy production can be positive or negative, but remains positive on time average, in accordance with generalized fluctuation–dissipation theorems^22^,^23^,^24^. The dissipation can also be locally very strong, sometimes over three orders of magnitude larger than the average injected power. The resulting distribution of dissipation intensity is strongly non-Gaussian, with very large tails (see Fig. 2).

Comparing with instantaneous maps of 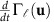 at the same scale, we see that besides areas of large dissipation, there are also areas of non-zero local rate of velocity circulation decay, which could be the footprints of singularities providing local source of circulation/vorticity, as conjectured by Eyink^16^. If we turn to the laminar case (case A, Fig. 1a,d,g), the resolution of our measurements over the whole flow is smaller than the relevant scale; thus, all scales are resolved. There are no clear dissipation structures in the map of *D*_*δx*_(**u**), which appears to be negative over the whole observation window and, on average, 3.5 times smaller than the viscous dissipation. The latter is also very small in that area, over one order of magnitude smaller than the total energy injection. In a similar way, we observe on Fig. 1d,g that both *D*_*δx*_(**u**) and 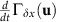 are very small at the centre compared with viscous dissipation and compared with their values for the two other (turbulent) flows. This is suggestive of the idea that the contribution of possible inertial dissipation plays a more important role at high Reynolds numbers, while viscous effects decrease. For case A, if the energy balance is performed over the whole experiment, the viscous dissipation accounts for all of the injected power and supersedes, by two order of magnitudes, the estimates of inertial dissipation. We also see by comparing Fig. 1b with Fig. 1e and Fig. 1c with Fig. 1f that areas of high viscous dissipation tend to be correlated with areas where strong inertial dissipation are localized.

**Figure 3:**
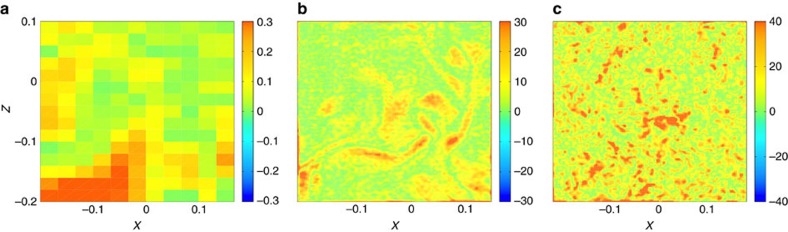
Maps of vorticity. Maps of the norm of the dimensionless *y*-component of the vorticity, 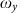, computed on the plane of measurement of our SPIV setup. (**a**) Case A, (**b**) case B and (**c**) case C. All the quantities are made dimensionless using *R* and (2π*F*)^−1^ as units of length and time.

To see whether the structures on Fig. 1d–f are located in areas of high vorticity, we may compare them with maps of vorticity magnitude^17^. In our case, we have only access to the *y* component of vorticity, 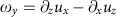 at the resolution scale, whose magnitude is displayed in Fig. 3 for the three cases described in Table 1.

Comparing Fig. 3b with Fig. 1e we find an overall agreement between the vorticity and dissipation map. However, we see that some structures in the *D*_*δx*_(**u**) field are not mirrored in the vorticity field, and that the agreement is worse for case A and C, showing that the link between vorticity and inertial dissipation might be restricted only to turbulent flows, when dissipative scale is resolved.

### Extreme events in the inertial dissipation estimates

**Figure 4:**
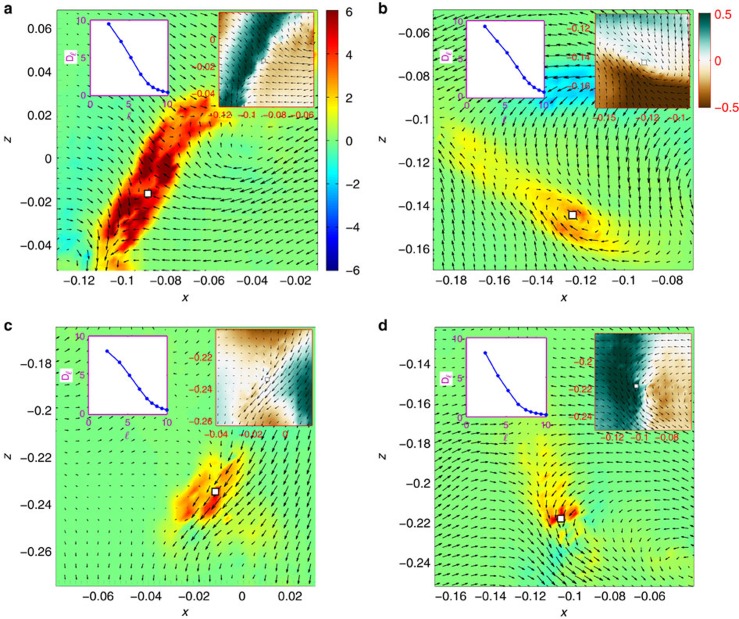
Types of extreme events. Four types of structures found from the extreme events (1,000 times the mean) of inertial dissipation estimates *D*_*δx*_(**u**) in case B, where Kolmogorov scale is resolved. (**a**) Front, (**b**) spiral, (**c**) jet and (**d**) cusp. Main figures: spatial maps of dimensionless magnitude of *D*_*δx*_(**u**), with arrows showing in-plane velocities around each extreme event. Common colormap shown in **a**. *D*_*δx*_(**u**) is normalized by *R*^2^(2*πF*)^3^ and positions are normalized by *R*. Right insets: the three-component velocity fields (*u*_*y*_ in colours) around each event (in units of 2π*RF*). All right insets share the colourmap in **b**. Coordinate axes are right handed. Left insets: *D*_*δx*_(**u**), averaged over a circle (of 21 points) around the peak (non-dimensionalized by *R*^2^(2*πF*)^3^) as a function of ℓ (non-dimensionalized by *η*) at the peak of each event.

To focus on the extreme events and to characterize them, we restrict our analysis to very intense events that are locally responsible for very large *D*_*δx*_(**u**). We harvest from case B (the turbulent resolved case) only those structures having *D*_*δx*_(**u**) of 1,000 times higher than its space-time average, corresponding to very rare extreme events. Out of 30,000 images, we found only 28 events, corresponding to probability of <1 in 50,000 (based on ratio of areas). Examples of these events are shown in Fig. 4. By observing the local velocity around these 28 events, we are able to classify them into 4 main types:

Fronts (Fig. 4a), where the velocity field shows two regions of very different velocities separated by a clear boundary along which the extreme event lies. In the frame of reference moving with the peak of the event, the in-plane velocities typically display a shock-like pattern. This type of structures is the result of two blobs of fluids, initially well-separated in space and velocity, being brought to close distance. In this sense, it is reminiscent of the fronts found in studies of turbulent mixing of passive scalar^25^. Similar patterns could also be found in weather patterns (for example, cold fronts). Many of these events also show velocity patterns such as in a saddle point (where fluid flows inwards on one axis while escaping on another) as can be seen in the periphery of Fig. 4a. In general, fronts and saddles belong to the same causal family in the sense that two blobs of fluid are mutually colliding and thus escape in other directions. These events are the most frequent, representing 21 events, that is, 75% of the cases. The inertial dissipation of most of these events (except two) increases without sign of saturation with decreasing scale, corresponding to a local exponent *h*<1/3 (otherwise *h*=1/3).

Spirals (Fig. 4b), where the in-plane velocity has a spiral structure. The inertial dissipation increases without saturation, corresponding to a local exponent *h*<1/3. This type of events was found three times. All these events are converging spirals consistent with the scenario of vortex stretching in the out-of-plane direction.

Jets (Fig. 4c), where the in-plane velocity has a strong narrow jet and the peak is found near the edge where there is strong shear. Another common feature is the complex profile of the out-of-plane velocity. These are suggestive of further breakdown energy by instability at certain location of fronts in the flow. The inertial dissipation increases without saturation, while showing possible saturation in another case. This type of events was only found two times.

Cusps with helicity (Fig. 4d), where the velocity field in the in-plane displays a horse-shoe-like structure, whereas the out-of-plane velocity profile is clearly distinct across the hypothesized cusps. These are events that seem incompatible with the above categories. They have features suggestive of the velocity field generated by a vorticity line motion with a local cusp and axial motion (see Supplementary Note 3). Such vorticity pattern has been frequently observed in numerical simulation of vortex lines reconnection^26^ and has even been suggested to be at the origin of the *k*^−5/3^ turbulent spectrum^27^. On the other hand, as shown by Danchin^28^, the velocity field generated by a cusp-like local vorticity patch is still regular, so that such a simple model might not be sufficient to explain our observations. The inertial dissipation around cusp events increases without saturation, corresponding to a local exponent *h*<1/3. They represent only two events.

## Discussion

We characterize, in our experiments, the topology of extreme events of inertial dissipation estimated at the dissipative scales of turbulence. Our results provide a further indication of the non-trivial structures of sub-Kolmogorov flows, complementary to previous studies based on scaling studies of dissipative intermittency, for example, see Sreenivasan^29^. We show that extreme inertial dissipation events are associated with the existence of velocity fronts, saddle points, spirals, jets and, in some cases, suggestive of cusps. These kinds of topologies are typically associated with special configurations of eigenvalues of the velocity strain tensors around critical points of flow patterns. At such points, it is often the case that lagrangian trajectories cross^30^, which would make these extreme events possible locations of shock-like singularities. In any case, the flow topology around the extreme inertial dissipation events is different from the usual flow topology associated with viscous dissipation. For instance, Moisy and Jimenez^31^ used box counting to study the fractal structure of regions of intense vorticity and energy dissipation in a direct numerical simulation of isotropic turbulence. Their work suggests that the geometry of the regions of intense dissipation resemble sheets or ribbons. This suggests that inertial dissipation and viscous dissipation are two different processes, at least down to the dissipative scale.

Another interesting observation is that extreme events of inertial dissipation provide significant local contributions to energy balance at the Kolmogorov scale, regardless of whether the energy lost pertaining to these events is eventually dissipated by singularities or by viscosity at yet smaller scales. This suggests that Kolmogorov scale is not the only characteristic scale for dissipation. This seemingly surprising conjecture is in fact compatible with the multi-fractal picture of turbulence, which predicts that for a given flow singularity of exponent *h*, there is a specific dissipation scale^32^ *η*_*h*_ scaling like *Re*^−1/(1+h)^. For *h*=1/3, we recover the classical Kolmogorov scale *η.* For the case with *h*<1/3, we have *η*_*h*_<*η*, so that the dissipation occurs at much smaller scale than the Kolmogorov one. Our findings are therefore compatible with the multi-fractal picture of turbulence, if the extreme events of inertial dissipation are the footprints of singularities of exponent *h*<1/3, as suggested in Kuzzay *et al*.^17^.

Whether this interpretation is valid or not is still debatable, as we have no means to follow the inertial dissipation down to ℓ=0, as required by the mathematical theorem^15^. To unambiguously distinguish between the possibilities of whether the energy contained in these extreme events is eventually dissipated by non-viscous mean or otherwise, one may need to resolve the flow down to the kinetic limit and track their evolution in time until they fully dissolve, which represents a experimental challenge for future works.

Perhaps a more immediate practical question one could ask is: knowing the significance of such extreme events even at the dissipative scales, how could one truncate models and simulations at tractable hydrodynamic scales with the correct physics reflecting their properties? In compressible fluid dynamics, these kinds of questions are usually addressed in relation with the building of a singularity through shock formation. In these cases, it has been common practice starting with von Neumann and Richtmyer^33^, to select physically admissible solutions and ensure the stability of numerical schemes via the introduction of an appropriate numerical viscosity^34^. Our results suggest that the same kind of procedure should also be introduced in incompressible numerical simulations, to account for extreme events of inertial dissipation that are not captured at the model resolution scale.

## Methods

### The choice of von Kármán swirling flow

As inertial dissipation is expected to originate from flow singularities, we focus on a geometry where lack of smoothness is not forbidden by mathematical theorems. This motivates our choice of an experimental set-up providing a turbulent flow with statistical axisymmetry. This kind of geometry has attracted interest from many works on the regularity of INSE^35^,^36^,^37^,^38^,^39^,^40^,^41^, where it was shown that the regularity properties of the axisymmetric Navier–Stokes equations heavily depend on the intensity of the swirl component of the flow *υ*_φ_ and its variation with respect to the distance from the rotation axis. When the swirl is zero, Ladyzhenskaya^35^ proved that the flow is smooth at all times. When the swirl is non-zero, the regularity can also be proven for finite time, in a domain excluding the symmetry axis^38^. In our experiment, we therefore currently concentrate our measurements on a domain including the symmetry axis and generate turbulence in a vertical cylinder of height *H* and radius *R* filled with water, and stirred by two coaxial, counter-rotating impellers (von Kármán flow) providing energy and momentum flux at the upper and lower end of the cylinder. The resulting flow is statistically axisymmetric, with a time-averaged velocity consisting of a swirl (toroidal flow) 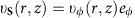 and a poloidal flow 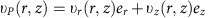, where (*r*, φ, *z*) are the cylindrical coordinates and (e_r_, e_*φ*_, e_z_) the corresponding unit vectors^42^. The ratio *υs*/*υp* is controlled by the impellers geometry. In the sequel, we focus on impellers such that *υs*/*υp=*2.5. The impellers are driven by two independent motors rotating at a frequency *F* and the experiment is thermalized at a temperature *T*≈20 °*C*.

### Torque and rotational frequency measurements

Torque (global) measurements at each impeller are performed with SCAIME technology and provide values over the kHz range of *C*_1_ and *C*_2_, being respectively the torque applied to the bottom and top shafts. They are calibrated using measurements at different mean frequencies, so as to remove spurious contributions from genuine offsets or mechanical frictions. From this, we compute the injected power necessary to maintain our turbulent flow in a statistical stationary state as *P*=2*π*(*C*_1_*F*_1_+*C*_2_*F*_2_), where *C*_1_ and *F*_1_ are the torques and the frequencies at the two impellers, respectively. To get a meaningful comparison between different impellers, we further renormalize the injected power by *ρ*R^5^(2*πF*)^3^, where *ρ* is the fluid density, *F*=*F*_*1*_=*F*_*2*_ (exact counter-rotating regime) and *R* is the radius of the cylinder.

### Particle image velocimetry

The typical size of the particles used in the PIV measurements is a few tens of micrometres and their density is 1.4. Two cameras take 30,000 successive pictures of the flow at a 15-Hz framerate. The resolution of our camera frame is 1,600 × 1200 pixels and the reconstruction is done using peak correlation performed over 50% overlapping windows of size 16 to 32. As a result, we get measurements of velocity field on a grid of approximate size 170 × 160 to 90 × 68 in a vertical plane containing the axis of symmetry (Oz), in a cylindrical system of coordinates. We performed two types of experiments: one with the cameras set at a distance such that their field of view covers the whole meridional plane. This set-up enables a global view of the flow and reaches a minimum grid step of the order of 200/68≈3 mm. In the second, we adapt lenses on the camera, to focus on a five times smaller field of view, of the order of 40 × 30 mm. Increasing the number of particles in the flow and using overlapping windows of size 16 × 16, we are then able to reach a minimum grid step of the order of 40/160≈0.25 mm. The continuity between the two types of measurements can be checked by degrading the resolution of the zoomed picture using overlapping windows of 32 × 32 or 128 × 128 for the velocity reconstruction. This last case corresponds to the velocity field obtained without lenses, with a velocity field reconstructed using windows of size 16 × 16.

The total acquisition time is ∼10 min to 2 h, that is, one or two orders of magnitude longer than the characteristic time of the slowest patterns of the turbulent flow. Fast scales are statistically sampled.

The velocity fields are non-dimensionalized using a typical velocity *V*_0_=2*πR*(*F*_1_+*F*_2_)/2 based on the radius of the cylinder and the rotation frequencies of the impellers. The resulting velocity fields are windowed so as to fit to the boundaries of the flow and remove spurious velocities measured in the impellers and at the boundaries. We apply a local filter (based on velocities of nearest neighbours) to remove isolated spurious vectors. Typically, ∼1% of the data are changed by this processing.

### Estimation of dissipation and circulation production rates

With our velocity fields, we can compute the velocity increments 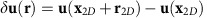, From this, we define two scale dependent scalar functions: the local energy dissipation rate *D*_ℓ_(**u**)^15^:


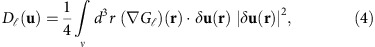


where *ν* is a full disk and the local rate of velocity circulation decay^16^:


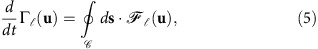


where


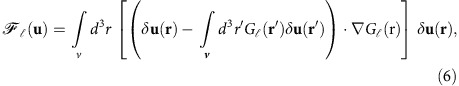


C being any contour advected by the fluid and *G*_ℓ_ is a spherically symmetric function of r given by^17^:


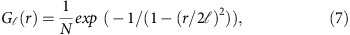


where *N* is a normalization constant such that 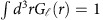.

In addition, we may also compute the local rate of viscous dissipation at the resolution scale, given by:


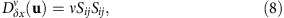


where 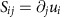. In the present case, we are missing some components of the viscous dissipation. Incompressibility condition provides *S*_22_=−*S*_11_−*S*_33_. We have also used statistical axisymmetry to replace *S*_21_ by *S*_12_ and *S*_23_ by *S*_13_. We have checked that this last hypothesis does not change the topology of the local maps of dissipation, but changes the time-average, hopefully accounting for the missing dissipation due to plane projection.

### Data availability

The data that support the findings of this study are available from the corresponding author upon request.

## Additional information

**How to cite this article:** Saw, E.-W. *et al*. Experimental characterization of extreme events of inertial dissipation in a turbulent swirling flow. *Nat. Commun.* 7:12466 doi: 10.1038/ncomms12466 (2016).

## Supplementary Material

Supplementary Information Supplementary Figures 1-8, Supplementary Notes 1-3 and Supplementary References.

## Figures and Tables

**Table 1:** Parameter space describing the 3 cases considered in this paper.

**Case**	***F* (Hz)**	***Re***	***η*** **(mm)**	***δx*** **(mm)**	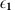	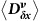	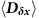
A	2	149	4.3	3.4	0.088	0.007	<0.0001
B	1.2	6 × 10^3^	0.32	0.24	0.049	0.07	0.007
C	5	3 × 10^5^	0.02	0.24	0.046	0.008	0.03

*δx* is the grid spacing of our measurements and 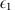 is the dimensionless injected power (in units of *R*^2^(2π*F*)^3^), averaged over the whole volume of the experiment, measured using torque meter. 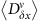 is the space-time average of the viscous dissipation measured from stereoscopic particle image velocimetry system data in a region of 4 × 4 cm^2^ localized at the centre of our experiment and 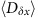 is the dimensionless space-time average of the inertial dissipation in the same region (all in units of *R*^2^(2π*F*)^3^).
