# Supplementary material for: Experimental characterization of extreme events of inertial dissipation in a turbulent swirling flow
Source: Nat Commun. 2016 Aug 31;7:12466. doi: 10.1038/ncomms12466 (PMC5013646; doi:10.1038/ncomms12466)
Supplement: Supplementary Information — Supplementary Figures 1-8, Supplementary Notes 1-3 and Supplementary References. [file ncomms12466-s1.pdf]

1 Supplementary Figures

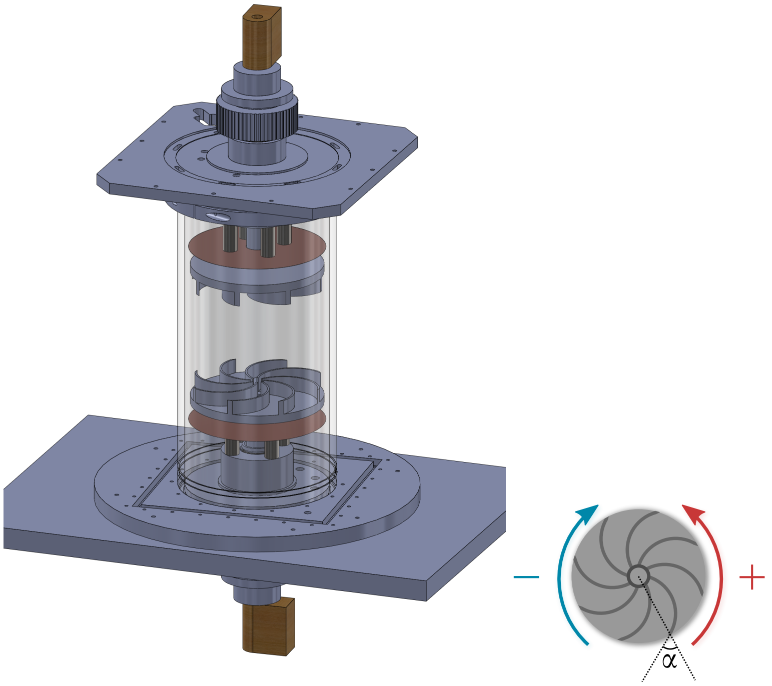

Supplementary figure 1: ***Experimental setup.*** *Picture of the experimental set-up and of an impeller. The notations for the two forcing conditions are defined along with the angle* α *characterizing the curvature of the blades.*

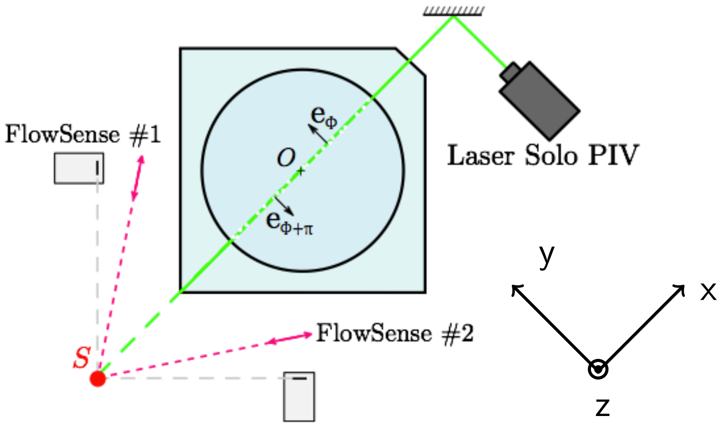

Supplementary figure 2: ***Imaging Setup.*** *Schematics of the PIV set-up viewed from above. A laser lights micrometer sized particles in a meridional plane, while two cameras take successive snapshots of the flow. This allows us to get the three components of the velocity field in the lit meridional plane.*

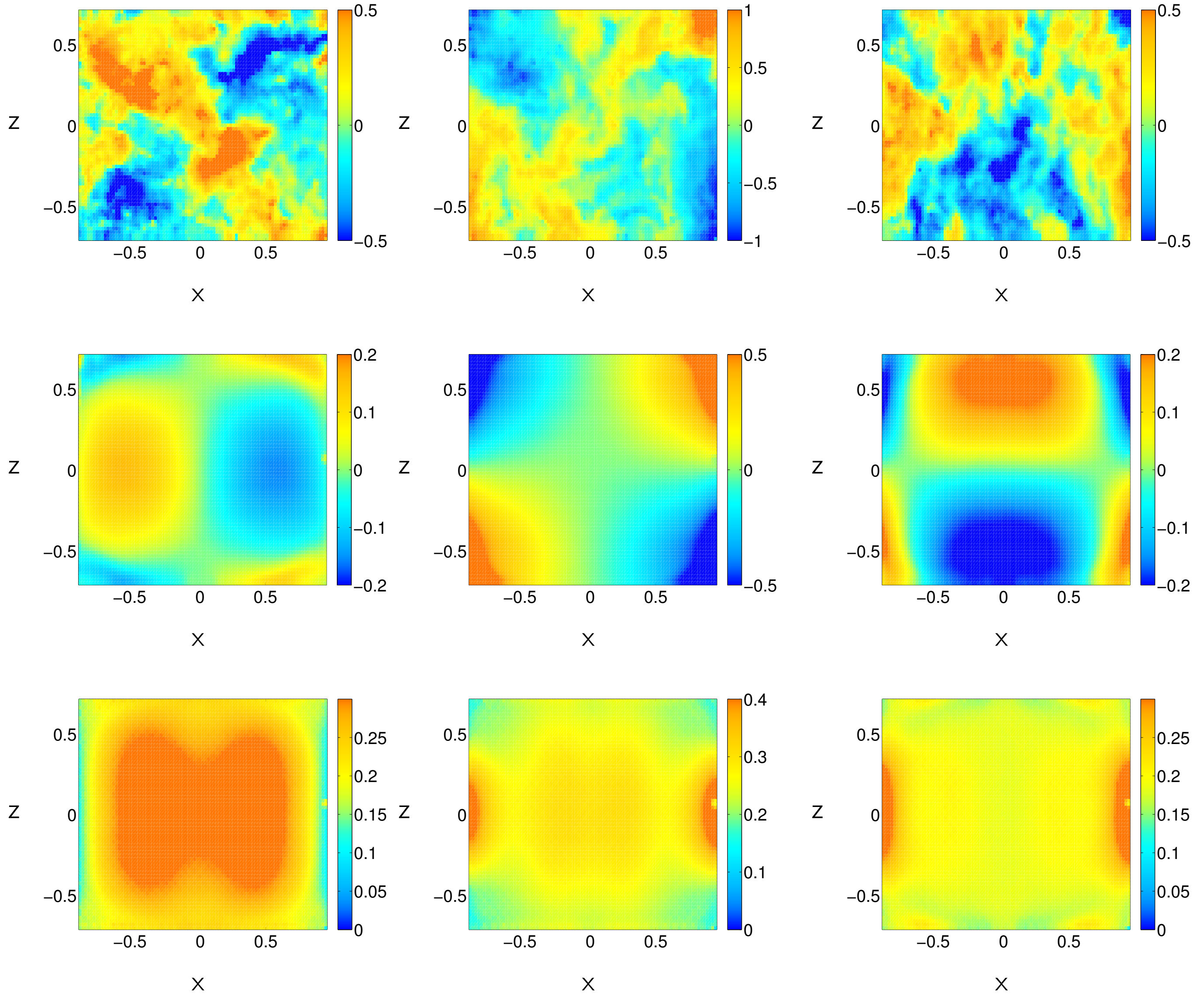

Supplementary figure 3: ***Mean flow and fluctuations.*** *Maps of the instantaneous (top panels) and mean (middle panels) flows along with the standard deviation (bottom panels) at each measurement points (same flow as in Fig. 1-4). Left column:* Vx. *Middle column:* Vy. *Right column:* Vz. *We see that the instantaneous flow is highly disordered, while the mean flow and the standard deviation have well defined structures. Mesurements made at* $Re \approx 3 \times 10^5$.

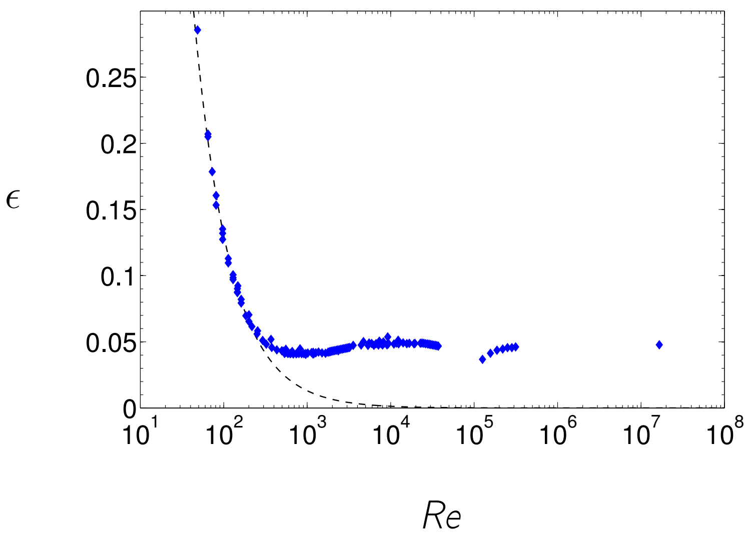

Supplementary figure 4: ***Mean injected kinetic power.*** *Global dimensionless injected power per unit mass* ϵ *(blue dots) in the stationary symmetric regime as a function of the Reynolds number* Re*. The dotted line represents the fit* $\epsilon \propto Re^{-1}$*. This figure has been adapted from Fig. 3 in* [5]*. The point at* $Re \approx 10^7$ *was measured using normal liquid helium in the SHREK experiment while all the other points have been obtained using water or glycerol.*

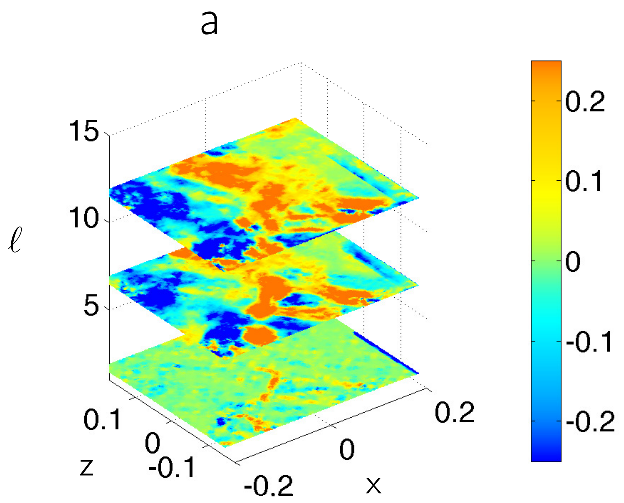

Supplementary figure 5: ***Variation of inertial energy dissipation with lenght-scale.*** *Maps of the instantaneous dimensionless coarse-grained inertial energy dissipation* $D_\ell(\mathbf{u})$ *as a function of scale* ℓ *for a flow at* $Re \approx 3 \times 10^5$. *a) Maps of* $D_\ell(\mathbf{u})$ *at three different scales. b) Maps of* $D_\ell(\mathbf{u})$ *at different scales, along a line going through a peak in inertial dissipation. The colors code* $D_\ell(\mathbf{u})$. *The scale is expressed in units of the grid step:* $0.25\ mm$.

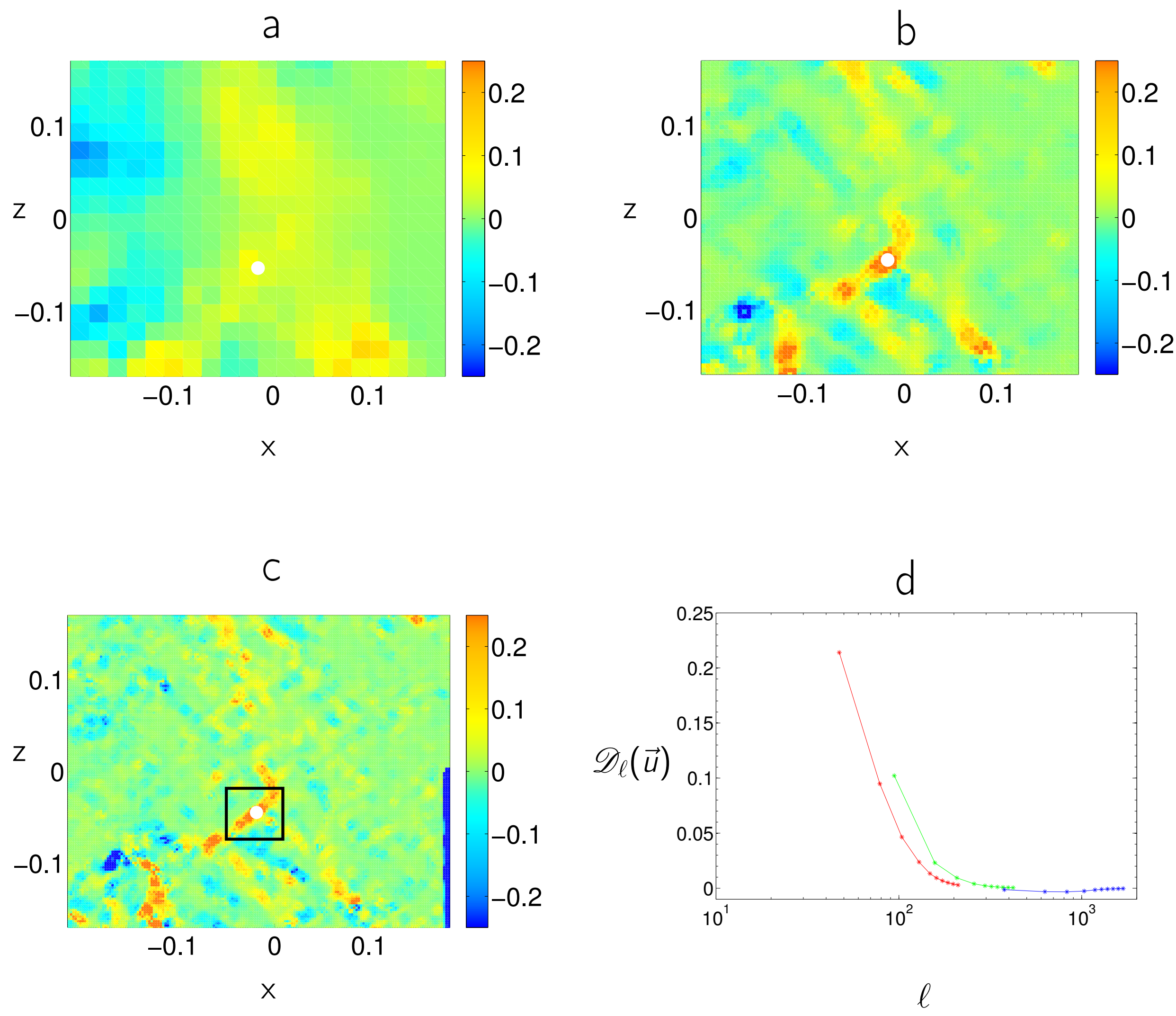

Supplementary figure 6: ***Inertial energy dissipation at different resolutions.*** *Maps of the instantaneous dimensionless coarse-grained inertial energy dissipation* $D_\ell(\mathbf{u})$ *at three different resolutions* ($2\delta x$)*: a) 4 mm (PIV window 128×128), b) 1 mm (PIV window 32×32) and c) 0.5 mm (PIV window 16×16). Figure d) shows* $D_\ell(\mathbf{u})$ *as a function of scale* ℓ*, at the "center" of the strong event identified by the white dot on the maps: blue: computed at the resolution* 4 *mm , green: computed at the resolution* 1 *mm; red: computed at the resolution* 0.5 *mm. The area inside the black square in figure c) is magnified in Fig. 7.*

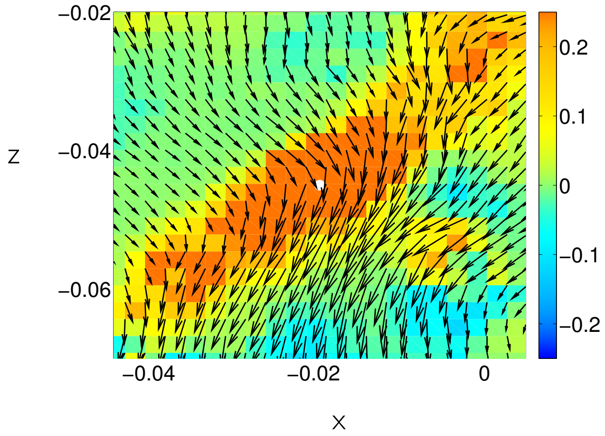

Supplementary figure 7: ***Local velocity around a strong event.*** *Local in-plane velocity around a strong inertial dissipation event.* The colors code the instantaneous dimensionless coarse-grained inertial energy dissipation $D_\ell(\mathbf{u})$, the arrows code the in-plane velocity. The region showed here corresponds to the area inside the black square of Fig. 6c). The white dot is at the same position as in Fig. 6. An abrupt change in the x-component of the velocity at this location can be observed.

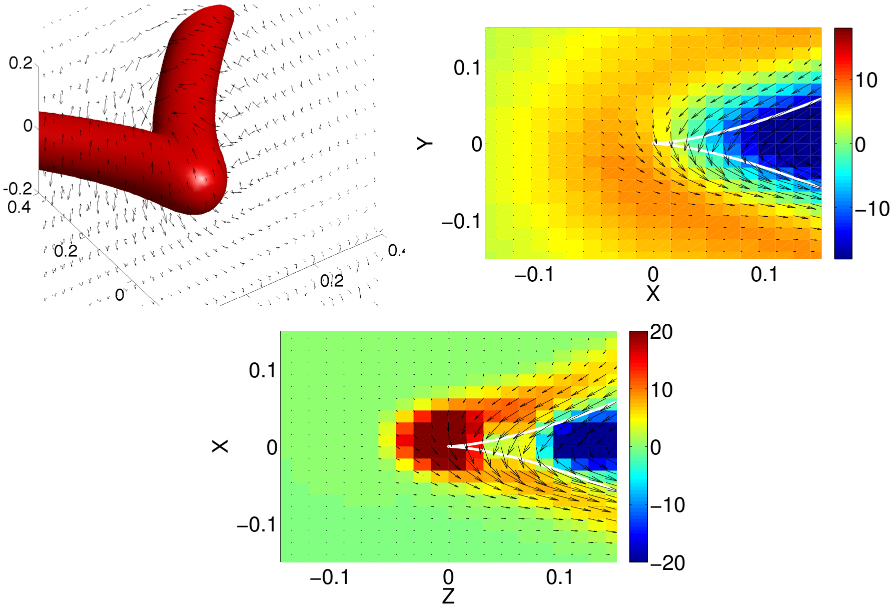

Supplementary figure 8: ***Simulated cusps.*** *(Color online) inertial dissipation near singularity generated by a vorticity cusp. Panel a: isocontour of vorticity showing the cusp at the origin. Panel b: velocity in the plane $Z = 0$ in the vicinity of the cusp. The white line is the location of maximum vorticity in that plane. The color code the out-of-plane velocity. The arrow codes the in-plane velocity. Panel c: inertial dissipation computed using the velocity field of Panel b. The color codes de inertial dissipation, while the arrow code the local in plane velocity. Units are arbitrary.*

2 Supplementary Notes

Supplementary Note 1: The experimental set-up Our von Kármán flow is generated by two counter-rotating impellers in a vertical cylinder (see Fig. 1). The radius of the cylinder is $R = 100\ mm$ and its height is $H = 240\ mm$. The impellers used in this paper are flat disks of diameter $185\ mm$, seperated by a distance $h = 180\ mm$ and fitted with 8 curved, radial blades of height $h_b = 20\ mm$. The curvature of the blades is characterized by an angle $|\alpha| = 72°$.

The impellers are driven by two independent motors which can rotate at frequencies up to typically $10\ Hz$. In our study, the motor frequencies have been set equal in order to get exact counter-rotating regime. The curvature of the blades allows to choose between two different forcing conditions in order to generate flows with different statistical properties. Here, we choose to rotate the impeller in the (-) direction defined on Fig. 1, to get $\alpha = -72°$

Our experimental set-up allows for both global and local flow diagnostics. Local velocity measurements are performed using Stereoscopic Particle Image Velocimetry (SPIV) in the stationary regime (see Fig. 2). The particles we have used have a size of a few tens of micrometers and their density is 1.4. A laser of wavelength 532 nm is used to light a meridional plane while two cameras of resolution 1600x1200 pixels, set at $45°$ with respect to that plane, take successive snapshots of the flow. Then, the velocity field is reconstructed using peak correlation performed over 50% overlapping windows of size 16 to 32 pixels. As a result, we get instantaneous snapshots of the three components of the velocity field on a grid of approximate size 90×70 (see Fig. 3).

Typical maps of the instantaneous (top panels) and time averaged (middle panels) velocity fields for the global experiments are provided on Fig. 3, along with maps of the standard deviation (bottom panels) of the three components of the velocity at each grid points at $Re \approx 3 \times 10^5$. The statistics for these maps have been obtained from 3×10^4 instantaneous snapshots. We observe that the instantaneous velocity fields are highly disordered contrary to the mean flow and the standard deviation which have well defined structures.

Along with local measurements, global diagnostics can be obtained. The torque applied to the top and bottom shafts are monitored using SCAIME technology, which allows us to measure the total power injected by the impellers into the flow (see Fig. 4). The calibration procedure, along with several other details on the experimental set-up may be found in [2,3] and references therein. Fig. 4 presents the global injected power per unit mass ϵ as a function of the Reynolds number Re (blue rhombi). It can be seen that at low Reynolds numbers (i.e $Re < 200$), ϵ decreases as Re^{-1} (dotted line). However, when Re becomes greater than 200, the flow becomes chaotic and a discrepancy between the experimental measurements and the Re^{-1} law appears. For very large Reynolds number, $Re > 10^5$, dissipation rate, ϵ, becomes constant [4] ($\epsilon \approx 0.046$).

Supplementary Note 2: Examples of measured inertial dissipation fields. An example of variation of $D_\ell(\mathbf{u})$ as a function of the scale ℓ and position $\mathbf{x}$ is provided in Fig. 5 for an instantaneous velocity field at $Re \approx 3 \times 10^5$. As the scale ℓ is decreased, the $D_{2D}(\mathbf{u})$ does not vanish, but instead points towards localized points which we identify as strong inertial dissipation event with $h \leq 1/3$.

For this computation, we have used a spherically symmetric function of x given by:

$$G_\ell(r) = \frac{1}{N} \exp(-1/(1-(r/(2\ell)^2))), \tag{1}$$

where N is a normalization constant such that $\int d^3 r G_\ell(r) = 1$. According to [1], the results should not depend on the choice of this function, in the limit $\ell \to 0$.

To estimate the scaling range of the extreme event, we have performed the computation of $D_\ell(\mathbf{u})$ at different resolutions, using different averaging windows to reconstruct the velocity flow from the same image. An example is provided in Fig. 6. One sees that, as the resolution is increased, the region of elevated $D_\ell(\mathbf{u})$ becomes sharper and sharper, but globally remains at the same location (emphasized by the white dot). On the other hand, the plot of $D_\ell(\mathbf{u})$ at this location (Fig. 6d) as a function of ℓ shows that there is a continuity between the measurements. For this event, $D_\ell(\mathbf{u})$ is slowly varying at large scale, suggestive of a flow sturture with $h \approx 1/3$ and then increases at the smallest scales. This is corroborated by a local plot of the in-plane velocity field around the event (Fig. 3). One clearly observes a front-like structure of the velocity field at this location.

This study is however only performed at scales larger than about 10 times the dissipative scale. Similar structures at the resolution scale is provided in the main part of this paper.

Supplementary Note 3: Simulation of dissipation around a cusp singularity We have simulated an artificial vorticity line with a cusp $\boldsymbol{\omega}(\mathbf{x})$ on a 64^3 grid (Fig. 8-a). and computed the associated velocity field $\mathbf{v}_{BS}$ using Biot-Savart law. To obtain a non-zero velocity along the vortex

line, we then consider the velocity $\mathbf{v} = \mathbf{v}_{BS} + \alpha\boldsymbol{\omega}$ (where α is the helicity). Its structure near the cusp in a plane parallel to the vortex line is provided in Fig. 8-b. We then computed the function $D_{\delta x}(\mathbf{u})$ in the same plane, which is provided in Fig. 8-c. This singularity map is also similar to the singularity map we observe in our experiment.

3 Supplementary References

1. Duchon, J., Robert, R. Inertial energy dissipation for weak solutions of incompressible Euler and Navier-Stokes equations. *Nonlinearity* **13,** 249255 (2000).

2. Cortet, P.-P. et al., Normalized kinetic energy as a hydrodynamical global quantity for inhomogeneous anisotropic turbulence. *Phys. Fluids* **21,** 025104 (2009).

3. Saint-Michel, B., Daviaud, F., Dubrulle, B. A zero-mode mechanism for spontaneous symmetry breaking in a turbulent von Kármán flow. *New J. Phys.* **16,** 013055 (2014).

4. Ravelet, F., Chiffaudel, A., Daviaud, F., Supercritical transition to turbulence in an inertially-driven von Kármán closed flow. *J. Fluid Mech.* **601,** 339-364 (2008).

5. Saint-Michel, B. et al. Probing quantum and classical turbulence analogy in von Kármán liquid helium, nitrogen, and water experiments. *Phys. Fluids* **26,** 125109 (2014).
